# Osteoporosis in Pediatric Orthopaedics: Congenital Diseases, Medical Management, and Perioperative Considerations

**DOI:** 10.1007/s11914-026-00985-y

**Published:** 2026-10-06

**Authors:** Aki S. Puryear, Laura E. Bess, Linda A. DiMeglio

**Affiliations:** 1 Department of Orthopaedic Surgery: Division of Pediatric Orthopaedics, Indiana University, Indianapolis, US; 2 Department of Pediatrics: Division of Pediatric Endocrinology and Diabetology, Indiana University, Indianapolis, US

**Keywords:** Pediatric osteoporosis, Osteogenesis imperfecta, Bisphosphonates, Bone mineral density, Neuromuscular scoliosis, Perioperative management

## Abstract

**Purpose of Review:**

Pediatric osteoporosis is a heterogeneous group of primary and secondary disorders that creates challenges for orthopaedic surgeons. This review summarizes evidence on conditions commonly encountered in pediatric orthopedic practice, medical management strategies, and perioperative care for children with bone fragility.

**Recent Findings:**

Diagnostic approaches have been evolving toward integrating fracture characteristics, skeletal risk factors, and bone mineral density (BMD). A single low-trauma vertebral compression fracture is now sufficient for diagnosis of osteoporosis in children regardless of BMD. Intravenous bisphosphonates remain first-line therapy, with recent meta-analyses confirming consistent BMD gains and, observationally, a reduction in fracture risk. Bisphosphonates are generally given to children who have sustained low-trauma vertebral fractures and considered for other children with recurrent long bone fractures with low BMD. Early intervention with bisphosphonates may also be warranted for children with high-risk diseases, such as Duchenne’s Muscular Dystrophy (DMD).

Biologic agents are emerging in clinical trials and select specialized use, but further research is needed to understand their role in pediatric osteoporosis. Denosumab carries a substantial risk for rebound hypercalcemia in children with osteogenesis imperfecta (OI), limiting its use to selected indications. Preliminary reporting from a large trial of anti-sclerostin therapy in children with osteogenesis imperfecta suggests significant BMD increases while fracture-reduction endpoints were not met, underscoring the limitations of BMD as a surrogate in pediatric bone fragility trials.

**Summary:**

Providing optimal care for this unique group of patients requires multidisciplinary coordination amongst orthopaedic surgeons, endocrinologists, geneticists, and rehabilitation specialists. Evidence-based management includes preoperative bone health evaluation, procedure-specific modifications in fragile bone, nutritional optimization, and coordinated bisphosphonate timing.

## Introduction

Pediatric osteoporosis is defined by the International Society of Clinical Densitometry (ISCD) as either a clinically significant fracture history together with a bone mineral density (BMD) Z-score less than or equal to -2.0, or one or more vertebral compression fractures in the absence of local disease or high-energy trauma, regardless of BMD [1, 2]. Unlike adult osteoporosis, which largely reflects age-related bone loss, pediatric osteoporosis arises from genetic conditions, chronic disease of childhood, and/or impaired bone mass accrual during growth, a critical window for peak bone mass acquisition.

For orthopaedic surgeons, pediatric osteoporosis is important not only because it increases fracture risk, but because it directly affects implant choice, fixation strategy, deformity correction, rehabilitation, and perioperative counseling [2–4]. Contemporary diagnostic thinking emphasizes the clinical context of each fracture, including its location, mechanism, recurrence, and associated risk factors, rather than relying solely on fracture counts or densitometry in isolation [1]. Although DXA-derived BMD remains the most widely used evaluative metric, it incompletely captures bone strength in children, particularly in conditions such as OI and neuromuscular disease, where children may sustain fragility fractures despite only modest reductions in BMD.

This review addresses three clinically relevant domains in pediatric orthopaedic practice: congenital and genetic diseases that predispose children to osteoporosis; current medical and multidisciplinary management; and the effects of pediatric osteoporosis on preoperative, intraoperative, perioperative, and postoperative care [4–7]. 

## Congenital and Genetic Diseases Predisposing to Pediatric Osteoporosis

### Osteogenesis Imperfecta

Osteogenesis imperfecta (OI) is the most common heritable skeletal fragility disorder and affects approximately 1 in 10,000 to 20,000 births [8, 9]. The traditional Sillence classification remains clinically useful, while more recent nosology groups OI according to the underlying disrupted molecular pathway [8, 9]. Most cases in genetically diverse populations are caused by dominant pathogenic variants in COL1A1 or COL1A2. However, numerous additional genes have been identified, and recessive forms represent an important subset in consanguineous populations [8, 9]. 

From an orthopaedic perspective, OI is associated with recurrent long-bone and vertebral fractures, progressive deformity, limb bowing, and scoliosis, often necessitating repeated surgical intervention [10, 11]. Fixation strategies in this population can be difficult owing to pre-existing deformities and diminutive stature in addition to poor bone quality. Fassier-Duval telescoping rods remain the preferred standard for growing children requiring intramedullary stabilization because they accommodate longitudinal growth and reduce repeated arthrotomy compared with earlier devices [10, 11]. 

### Neuromuscular Disorders

Several neuromuscular disorders commonly seen in pediatric orthopaedics are strongly associated with osteoporosis due to reduced mechanical loading, malnutrition, delayed puberty, chronic inflammation, and medication effects [4, 12, 13]. Children with severe cerebral palsy, especially Gross Motor Function Classification System (GMFCS) levels IV and V, often have markedly low BMD and are particularly prone to distal femur fractures and vertebral fractures due to their non-ambulatory status and high nutritional needs [4, 12, 13]. 

Duchenne muscular dystrophy (DMD) presents a dual challenge because progressive immobility coexists with glucocorticoid-associated bone loss [14–16]. Vertebral fractures are common in glucocorticoid-treated boys with DMD, and long-bone fractures may precipitate permanent loss of ambulation in those with borderline mobility [14–16]. Spinal muscular atrophy and Rett syndrome are also associated with low bone density, scoliosis, and fragility fractures that complicate surgical planning [14–16]. 

### Secondary and Iatrogenic Causes

Glucocorticoid-induced osteoporosis is the most common form of secondary pediatric osteoporosis and is encountered in children with rheumatic disease, leukemia, inflammatory bowel disease, nephrotic syndrome, and DMD [17, 18, 4]. Data from the Canadian Study of the Treatment of Pediatric Osteoporosis (STOPP) program showed that vertebral fractures are often present at diagnosis in acute lymphoblastic leukemia and may also develop early after glucocorticoid initiation in rheumatic disease, underscoring the need for baseline and interval spine surveillance in high-risk children [17, 18]. 

Idiopathic juvenile osteoporosis remains a diagnosis of exclusion characterized by prepubertal onset, metaphyseal and vertebral fractures, pain, and difficulty walking, with partial or complete remission often occurring around puberty [19]. Broader genetic testing has reduced the frequency of truly idiopathic cases by revealing monogenic causes of fragility in children previously classified as having idiopathic disease [19] Table1. 

**Table 1:** Conditions associated with pediatric osteoporosis in orthopaedic practice

Condition	Typical prevalence	Etiology	Key orthopaedic manifestations	Management highlights
Osteogenesis imperfecta	1:10,000–20,000 births [4]	COL1A1/COL1A2; many additional dominant and recessive genes [20, 21]	Recurrent long-bone and vertebral fractures, limb deformities, scoliosis [4]	IV bisphosphonates, telescoping rods, spine surveillance, tailored fixation [4, 10, 11]
Cerebral palsy	About 2–3 per 1,000 births [4]	Reduced loading, anticonvulsants, nutrition/endocrine factors [22, 23]	Distal femur fractures, vertebral fractures, low BMD in nonambulatory children [4]	Vitamin D/calcium optimization, weight-bearing exercise/standers, fracture prevention, selective bisphosphonate use [4, 22]
Duchenne muscular dystrophy	About 1:3,500-6,000 male births [4]	DMD pathogenic variants; chronic glucocorticoid exposure [4]	Vertebral fractures, long-bone fractures, scoliosis, loss of ambulation after fracture [4]	Lateral spine monitoring, glucocorticoid optimization, and bisphosphonates when indicated [4]
Spinal muscular atrophy	About 1:6,000–10,000 births [4]	SMN1-related disease [4]	Low BMD, scoliosis, long-bone fragility [4]	Disease-modifying therapy plus bone health surveillance and fracture-directed treatment [4]
Rett syndrome	About 1:10,000 females [4]	MECP2-related disease [4]	Progressive scoliosis, low-trauma fractures [4]	Calcium/vitamin D optimization, selective bisphosphonate use, scoliosis planning [22]
Glucocorticoid-induced osteoporosis	Common in at-risk chronic disease populations [4]	Glucocorticoid exposure and underlying disease [4]	Vertebral fractures, low BMD, impaired bone accrual [4]	Steroid-sparing strategies when possible, vertebral fracture surveillance, and bisphosphonates for significant osteoporosis [4]

## Medical Management and Multidisciplinary Care

### Bisphosphonate Therapy

Intravenous bisphosphonates remain the cornerstone of medical management for children with clinically significant osteoporosis, particularly in OI and in secondary osteoporosis with vertebral fractures or recurrent fragility fractures [4, 24, 25]. Cyclic pamidronate and intermittent zoledronic acid have consistently improved lumbar spine BMD and vertebral morphology in pediatric studies, and recent meta-analysis confirms meaningful BMD benefits in secondary pediatric osteoporosis, even though most studies were not powered primarily for fracture outcomes [4, 24, 25]. 

Recent observational data also suggests a reduction in fracture rate with zoledronic acid in children and adolescents with primary and secondary skeletal fragility. However, these findings are derived primarily from cohort studies rather than large randomized fracture-endpoint trials [26]. Pediatric experience remains reassuring regarding osteonecrosis of the jaw, which has not been reported in children treated for osteoporosis. Similarly, atypical femur fractures appear uncommon and difficult to separate from underlying disease severity [4, 26]. 

### Denosumab: Selected use and Important Risks

Denosumab has been used in selected pediatric disorders, including OI type VI and RANKL-mediated lesions, because of its potent antiresorptive effect [27–29]. However, rebound hypercalcemia after discontinuation is a major limitation in children. In prospective comparative data from pediatric OI, approximately 31% of treated patients developed rebound hypercalcemia, and nearly half of those affected experienced hypercalcemic crisis [27]. At this time, Denosumab should not be considered first-line therapy for pediatric osteoporosis and should be reserved for carefully selected indications in expert centers with close biochemical monitoring and a planned transition strategy [27–29]. 

### Emerging Anabolic Therapies

Anabolic strategies are of major interest in pediatric bone fragility because they may improve bone size and quality in ways that differ from antiresorptive therapy [30, 31]. Setrusumab, an anti-sclerostin antibody, generated substantial interest after earlier studies showed favorable effects on bone parameters, but an Ultragenyx press release in December 2025 regarding the phase 3 ORBIT and COSMIC trials noted that these trials failed to meet their primary fracture-reduction endpoints despite significant BMD gains (further analyses and formal publication still pending) [32, 33]. These studies are especially important because they were designed around fracture outcomes rather than BMD alone, reinforcing that increases in BMD may not reliably predict clinically meaningful fracture reduction in pediatric primary osteoporosis [32, 33]. 

Teriparatide use remains investigational in OI. Clinical experience is largely limited to adults, where randomized trials have shown increases in BMD, although fracture outcomes appear variable. Pediatric research and use was initially limited by a black box warning, however this has been removed based on post-marketing surveillance evidence in adults that the drug does not increase osteosarcoma risk, although uncertainty remains about safety and skeletal effects in growing skeletons [30]. At present, sequential treatment models involving an anabolic phase followed by antiresorptive consolidation are conceptually attractive but not standard pediatric practice [30, 31]. 

### Nutrition and Rehabilitation

Adequate calcium, vitamin D, protein intake, mobility, and physical therapy remain foundational components of treatment for every child with osteoporosis or bone fragility [4, 22]. For children and adolescents at risk, many pediatric bone specialists target serum 25-hydroxyvitamin D levels of at least 30 ng/mL even though population-level recommendations for vitamin D sufficiency and rickets prevention are more conservative [4, 22]. Weight bearing interventions, including standers, are also critical for bone health. Nutritional screening and rehabilitation planning are particularly important before major reconstructive surgery, when malnutrition and prolonged immobilization can further compromise bone health and healing [22]. Additionally, ensuring calcium sufficiency before bisphosphonate infusions can mitigate the risk of post-infusion hypocalcemia [25]. For children and adolescents without identifiable vitamin D deficiency, guidelines on daily requirements are less clear [34, 35]. Table 2 details vitamin D supplementation by age group. Supplementation in infancy has strong consensus amongst specialty groups. Infants should receive at least 400 IU/day in order to prevent deficiency. For children and adolescents without identifiable vitamin D deficiency, guidelines on daily requirements are less clear. Individual risk factors and needs for vitamin D should be considered when recommending supplementation. For those with vitamin D deficiency, supplementation may be provided with amount generally corresponding to degree of deficiency.

**Table 2:** Vitamin D Supplementation Recommendations by Age

Age	Vitamin D Supplementation	Sources
Infants (0–12 m)	400 IU/day	Formula Vitamin D drops Maternal Vitamin D supplementation (6400 IU/day)
Children (1-9y) No risk factors Risk Factors	400 IU/day 600-1,000 IU/day	Diet Supplements
Adolescents (10-19y) No risk factors At risk for deficiency	400-2,000 IU/day 1,000–4,000 IU/day	Diet Supplements

### Multidisciplinary Care

The most effective model for management of pediatric osteoporosis is multidisciplinary and typically includes pediatric orthopaedics, endocrinology or nephrology, radiology, genetics, anesthesia, nutrition, and rehabilitation [4, 22, 23]. This coordinated approach is especially important when children require both long-term medical therapy and major orthopaedic procedures, because medication timing, imaging interpretation, and postoperative mobilization plans all influence outcome [4, 22, 23]. Ongoing monitoring by DXA requires attention to detect significant change and growth-related changes in bone size, rather than absolute BMD alone.

## Perioperative Considerations

### Preoperative Assessment and Optimization

Children with known or suspected osteoporosis who are being considered for elective orthopaedic surgery should undergo a systematic preoperative bone health assessment [1, 2, 4]. Laboratory evaluation commonly includes serum calcium, phosphorus, creatinine, PTH, alkaline phosphatase, albumin, and 25-hydroxyvitamin D. Children with delayed puberty and/or conditions that may result in hypogonadism may need additional laboratory studies, as delayed exposure to gonadal hormones can result in significant osteoporosis.

Imaging typically includes lumbar spine and total body less head DXA, with lateral distal femur DXA in some centers being a useful option in nonambulatory children [1, 2, 4]. Vertebral fracture assessment (VA) using lateral spine imaging or DXA-based VFA is increasingly recommended in high-risk populations, as many vertebral fractures are clinically silent yet diagnostic of osteoporosis.

Interpretation of pediatric densitometry requires Z-scores rather than T-scores, and adjustments for stature and/or delayed pubertal skeletal maturation are essential to avoid overdiagnosis [1, 2, 4]. In selected patients with neuromuscular scoliosis, quantitative computed tomography may add useful volumetric data for surgical planning when low bone density is likely to affect fixation strength or achievable correction [2, 3]. 

### Intraoperative Technique Modifications

Implant selection in pediatric osteoporosis should consider poor bone quality, pre-existing deformity, and preventing future fracture with implant positioning. For this reason, intramedullary implants are often preferred due to the ability to protect the entire involved bone. However, many patients with poor bone quality and associated deformities may require osteotomies to facilitate passage of these implants. In the pediatric population, skeletal immaturity presents an additional challenge. In long-bone surgery for OI, telescoping intramedullary implants remain the preferred strategy when anatomy permits because they provide longitudinal support while accommodating growth [10, 11]. Surgical technique should minimize additional bone injury by limiting heat generation, and using gentle handling throughout positioning and fixation [4, 10, 11]. Post-operatively, supplemental immobilization through bracing or casting may be considered.

Spinal fixation in low-BMD bone often requires increased attention to screw trajectory, fixation density, and adjunctive measures to improve purchase [36, 37]. Calcium phosphate cement is generally preferable to polymethyl methacrylate in children when augmentation is required because it is osteoconductive and integrates with bone. However, use must be individualized according to anatomy and indication [36, 37]. In neuromuscular scoliosis, fixation to the pelvis and broader construct planning often depend on pelvic obliquity, ambulatory status, and bone quality rather than diagnosis alone [36, 37]. 

### Anesthetic Considerations

Children with OI and DMD require specialized anesthetic planning [38]. In OI, careful positioning and airway management are important because fractures can occur with transfer and cervical manipulation. Certain paralytics, such as succinylcholine should be avoided because muscle fasciculations may precipitate fractures [38]. In DMD, succinylcholine and volatile anesthetic agents are avoided because of the risk of rhabdomyolysis and life-threatening hyperkalemia and arrhythmia. Preoperative cardiac and pulmonary assessment is mandatory in this patient population before major surgery [38] Table 3 provides an overview of common orthopaedic conditions associated with osteoporosis and perioperative considerations. 

**Table 3:** Perioperative considerations by condition

Condition	Preoperative priorities	Intraoperative modifications	Postoperative considerations
Osteogenesis imperfecta	DXA, vertebral imaging, calcium intake, vitamin D and mineral status, review of prior surgeries, consideration of bony deformities [2, 1, 22]	Telescoping rods when feasible, careful positioning, atraumatic technique, fixation augmentation when needed [4, 10, 11]	Early mobilization, coordinated bisphosphonate restart after fracture surgery, delayed restart after planned osteotomy until healing [4–6]
Cerebral palsy with severe immobility	Lateral distal femur DXA or alternative imaging, nutrition screening, contracture and positioning assessment [2, 22, 23]	Broader fixation strategy for low bone density, pelvic fixation when indicated, pressure and positioning precautions [4]	Early rehabilitation, nutrition support, and monitoring for vertebral and distal femur fragility [4, 22, 23]
Duchenne muscular dystrophy	Lateral spine surveillance, vitamin D, cardiac and pulmonary assessment, fracture history [4, 22]	Bone-quality-aware deformity correction; avoid succinylcholine and volatile anesthetics [38]	Respiratory support planning, early mobilization when safe, vertebral fracture-directed bone health treatment [4]
Glucocorticoid-induced osteoporosis	Baseline spine imaging, DXA, review of cumulative steroid exposure, and endocrine status [2–4]	Consider a stronger fixation strategy in severely osteoporotic bone [4]	Ongoing monitoring for vertebral fractures, optimize steroid-sparing therapy where possible [4]

### Bisphosphonate Timing Around Surgery

Perioperative bisphosphonate timing remains a practical issue for orthopaedic teams [4–6]. Available observational evidence and expert consensus suggest that bisphosphonates can generally be resumed about two to four weeks after fracture fixation without clear evidence of impaired healing. In contrast, planned osteotomies are often managed with a treatment-free interval of several months before and after surgery until radiographic healing is established [4–6]. More recent spine data in medically complex children with neuromuscular scoliosis suggest that preoperative zoledronic acid can be used safely in selected patients when coordinated carefully with the surgical team [5, 6]. 

## Future Directions

Recent therapeutic development has highlighted both the progress and the limits of current pediatric bone research [4]. The preliminary negative primary results in the ORBIT and COSMIC Setrusumab trials, despite favorable BMD effects, show that future studies must continue to prioritize fracture outcomes and patient-centered functional measures rather than relying on densitometric improvement alone [32, 33]. 

Other promising directions include improved vertebral fracture detection through standardized imaging approaches, wider access to genetic diagnoses and interpretations, and development of more precise measures of quantifying bone quality and microarchitecture beyond DXA alone, including peripheral quantitative CT and trabecular bone score measures [2–4]. These advances may ultimately allow clinicians to identify earlier and more precisely which children need preventive intervention, which are most likely to benefit from targeted therapies, and how to personalize surgical planning and implant selection in fragile bone [2–4]. 

## Conclusion

Pediatric osteoporosis sits at the intersection of endocrinology, genetics, rehabilitation, and orthopaedic surgery [4, 30, 31]. For pediatric orthopaedic surgeons, optimal care depends on recognizing the underlying cause of bone fragility, using standardized pediatric diagnostic criteria, integrating medical treatment with operative planning, and coordinating care across specialties. [1, 2,30, 31] Intravenous bisphosphonates remain the standard pharmacologic foundation for most children with clinically significant osteoporosis. At the same time, denosumab and emerging anabolic therapies should be approached cautiously and selectively until stronger pediatric safety and fracture-outcome data become available [27–33]. 

## Key References

- Ward LM, Weber DR, Munns CF, Hogler W, Zemel BS. A contemporary view of the definition and diagnosis of osteoporosis in children and adolescents. J Clin Endocrinol Metab. 2020;105(5):e2088-e2097. 
  - ○ This review provides the clearest modern framework for pediatric osteoporosis diagnosis and is particularly valuable for distinguishing fracture-based diagnosis from BMD-only interpretation.[1]
- Shuhart CR, Yeap SS, Anderson PA, et al. Executive Summary of the 2019 ISCD Position Development Conference on Monitoring Treatment, DXA Cross-calibration and Least Significant Change, Spinal Cord Injury, Peri-prosthetic and Orthopedic Bone Health, Transgender Medicine, and Pediatrics. J Clin Densitom. 2019;22(4):453-471. 
  - ○ This paper establishes the key pediatric densitometry positions used in clinical practice, including preferred DXA sites and interpretation principles.[2]
- Glorieux FH, Bishop NJ, Plotkin H, Chabot G, Lanoue G, Travers R. Cyclic administration of pamidronate in children with severe osteogenesis imperfecta. N Engl J Med. 1998;339(14):947-952. 
  - ○ This landmark study established intravenous bisphosphonate therapy as the foundation of medical management in severe OI.[24]
- Ward LM. A practical guide to the diagnosis and management of osteoporosis in childhood and adolescence. Front Endocrinol (Lausanne). 2023;14:1266986. 
  - ○ This is the most practical recent review for translating pediatric bone health principles into day-to-day diagnosis, treatment selection, and perioperative management.[4]
- Liu J, Lin X, Sun L, et al. Safety and efficacy of denosumab in children with osteogenesis imperfecta: the first prospective comparative study. J Clin Endocrinol Metab. 2024;109(7):1827-1836. 
  - ○ This study is pivotal because it quantifies the high risk of rebound hypercalcemia after denosumab discontinuation in pediatric OI.[27]
- Ultragenyx and Mereo BioPharma. Phase 3 ORBIT and COSMIC results for setrusumab in osteogenesis imperfecta. Company communication/press release, December 28, 2025. 
  - ○ These trial results are important because they demonstrated improved BMD without primary fracture reduction, challenging the use of BMD as a surrogate endpoint in pediatric OI trials.[32, 33]

## Data Availability

No datasets were generated or analysed during the current study.

## References

[1] Ward LM, Weber DR, Munns CF, Högler W, Zemel BS. A Contemporary View of the Definition and Diagnosis of Osteoporosis in Children and Adolescents. J Clin Endocrinol Metab. 2020;105(5):e2088–97. 10.1210/clinem/dgz294 PMC7121121 31865390

[2] Shuhart CR, Yeap SS, Anderson PA, et al. Executive Summary of the 2019 ISCD Position Development Conference on Monitoring Treatment, DXA Cross-calibration and Least Significant Change, Spinal Cord Injury, Peri-prosthetic and Orthopedic Bone Health, Transgender Medicine, and Pediatrics. J Clin Densitom. 2019;22(4):453–71. 10.1016/j.jocd.2019.07.001 31400968

[3] Weber DR, Boyce A, Gordon C, et al. The Utility of DXA Assessment at the Forearm, Proximal Femur, and Lateral Distal Femur, and Vertebral Fracture Assessment in the Pediatric Population: 2019 ISCD Official Position. J Clin Densitom. 2019;22(4):567–89. 10.1016/j.jocd.2019.07.002 PMC7010480 31421951

[4] Ward LM. A practical guide to the diagnosis and management of osteoporosis in childhood and adolescence. Front Endocrinol (Lausanne). 2023;14:1266986. 10.3389/fendo.2023.1266986 PMC10875302 38374961

[5] Tabaie SA, Muir RT, Schneider M, et al. A Comprehensive Review of Bone Health in a Child: From Birth to Adulthood. J Am Acad Orthop Surg. 2024;32(9):363–72. 10.5435/JAAOS-D-23-00853 38261781

[6] Sakka SD, Cheung MS. Management of primary and secondary osteoporosis in children. Ther Adv Musculoskelet Dis. 2020;12:1759720X20969262. 10.1177/1759720X20969262 PMC7649886 33224280

[7] Günay A, Turan S. Pediatric osteoporosis: An update. Trends Pediatr. 2024;5(4):105–15. 10.59213/TP.2024.202.

[8] Sillence DO, Senn A, Danks DM. Genetic heterogeneity in osteogenesis imperfecta. J Med Genet. 1979;16(2):101–16. 10.1136/jmg.16.2.101 PMC1012733 458828

[9] Panzaru MC, Caba L, Gorduza EV. Classification of osteogenesis imperfecta: Importance for prophylaxis and genetic counseling. World J Clin Cases. 2023;11(12):2604–20. 10.12998/wjcc.v11.i12.2604 PMC10198117 37214584

[10] Birke O, Davies N, Latimer M, et al. Experience with the Fassier-Duval telescopic rod: first 24 consecutive cases with a minimum of 1-year follow-up. J Pediatr Orthop. 2011;31(4):458–64. 10.1097/BPO.0b013e31821bfb50 21572286

[11] Nguyen CV, Makarewich CA, Poon SC, et al. Long-term Outcomes of Intramedullary Nails in Osteogenesis Imperfecta: Fewer Surgeries and Longer Survival Times with Telescoping Rods in Patients With Over Ten Years Follow-up. J Pediatr Orthop. 2025;45(1):51–5. 10.1097/BPO.0000000000002810 39210524

[12] Barbier V, Goeb V, Gouron R, et al. Bone health in children with severe cerebral palsy. Front Pediatr. 2023;11:1264111. 10.3389/fped.2023.1264111 PMC10716435 38094189

[13] Henderson RC, Lark RK, Gurka MJ, et al. Bone Density and Metabolism in Children and Adolescents with Moderate to Severe Cerebral Palsy. Pediatrics. 2002;110(1):e5. 10.1542/peds.110.1.e5 12093986

[14] Birnkrant DJ, Bushby K, Bann CM, et al. Diagnosis and management of Duchenne muscular dystrophy, part 2: respiratory, cardiac, bone health, and orthopaedic management. Lancet Neurol. 2018;17(4):347–61. 10.1016/S1474-4422(18)30025-5 PMC5889091 29395990

[15] Ward LM, Hadjiyannakis S, McMillan HJ, et al. Bone Health and Osteoporosis Management of the Patient With Duchenne Muscular Dystrophy. Pediatrics. 2018;142(Suppl 2):S34–42. 10.1542/peds.2018-0333E PMC6442478 30275247

[16] Singh A, Schaeffer EK, Reilly CW. Vertebral Fractures in Duchenne Muscular Dystrophy Patients Managed with Deflazacort. J Pediatr Orthop. 2018;38(6):320–4. 10.1097/BPO.0000000000000817 27328118

[17] Ward LM, Konji VN, Ma J. The management of osteoporosis in children. Osteoporos Int. 2016;27:2147–79. 10.1007/s00198-016-3515-9 27125514

[18] Halton J, Gaboury I, Grant R, et al. Advanced vertebral fracture among newly diagnosed children with acute lymphoblastic leukemia: results of the Canadian Steroid-Associated Osteoporosis in the Pediatric Population (STOPP) Research Program. J Bone Min Res. 2009;24(7):1326–34. 10.1359/jbmr.090202 PMC3890351 19210218

[19] DiMeglio LA. Pediatric Bone Disorders. In: Pescovitz OH, Eugster EA, editors. Pediatric Endocrinology: Mechanisms, Manifestations, and Management. Philadelphia: Wolters Kluwer Health; 2004. pp. 671–98.

[20] Jovanovic M, Marini JC. Update on the Genetics of Osteogenesis Imperfecta. Calcif Tissue Int. 2024;115(6):891–914. 10.1007/s00223-024-01266-5 PMC11607015 39127989

[21] Yu H, Zhang Y, Song C, et al. Pathogenic mechanisms of osteogenesis imperfecta: evidence for classification. Orphanet J Rare Dis. 2023;18:234. 10.1186/s13023-023-02849-5 PMC10411007 37559063

[22] Martins DS, Moreira A, Albuquerque G, et al. Nutrition considerations in pediatric surgical patients. Nutr Clin Pract. 2022;37(3):510–20. 10.1002/ncp.10855 35502496

[23] Canada NL, Mullins L, Pearo B, Spoede E. Optimizing Perioperative Nutrition in Pediatric Populations. Nutr Clin Pract. 2016;31(1):49–58. 10.1177/0884533615622639 26703960

[24] Glorieux FH, Bishop NJ, Plotkin H, Chabot G, Lanoue G, Travers R. Cyclic Administration of Pamidronate in Children with Severe Osteogenesis Imperfecta. N Engl J Med. 1998;339(14):947–52. 10.1056/NEJM199810013391402 9753709

[25] Dwan K, Phillipi CA, Steiner RD, Basel D. Bisphosphonate therapy for osteogenesis imperfecta. Cochrane Database Syst Rev. 2016;10:CD005088. 10.1002/14651858.CD005088.pub4 PMC6611487 27760454

[26] Stoffers AJ, Mancilla EE, Levine MA, et al. Clinical efficacy of zoledronic acid on fracture reduction in youth with primary and secondary skeletal fragility. J Clin Endocrinol Metab. 2025;110(7):2031–40. 10.1210/clinem/dgae661 PMC12187120 39324646

[27] Liu J, Lin X, Sun L, et al. Safety and Efficacy of Denosumab in Children With Osteogenesis Imperfecta: the First Prospective Comparative Study. J Clin Endocrinol Metab. 2024;109(7):1827–36. 10.1210/clinem/dgad732 PMC11180505 38198649

[28] Vanderniet JA, Szymczuk V, Högler W, et al. Management of RANKL-mediated disorders with denosumab in children and adolescents: a global expert guidance document. J Clin Endocrinol Metab. 2024;109(5):1371–82. 10.1210/clinem/dgad657 PMC11031248 38041865

[29] Seale E, Ochoa Molina M, Carsen S, et al. Mitigating the Denosumab-Induced Rebound Phenomenon with Alternating Short- and Long-Acting Anti-resorptive Therapy in a Young Boy with Severe OI Type VI. Calcif Tissue Int. 2023;112(5):613–20. 10.1007/s00223-023-01065-4 36867194

[30] Bandeira F, Lewiecki EM. Anabolic therapy for osteoporosis: update on efficacy and safety. Arch Endocrinol Metab. 2022;66(5):707–16. 10.20945/2359-3997000000566 PMC10118815 36382760

[31] Glorieux FH, Devogelaer JP, Durigova M, et al. BPS804 anti-sclerostin antibody in adults with moderate osteogenesis imperfecta: results of a randomized phase 2a trial. J Bone Min Res. 2017;32(7):1496–504. 10.1002/jbmr.3143 28370407

[32] Glorieux FH, Langdahl B, Chapurlat R, et al. Setrusumab for the treatment of osteogenesis imperfecta: 12-month results from the phase 2b ASTEROID study. J Bone Min Res. 2024;39(9):1215–28. 10.1093/jbmr/zjae112 PMC11371902 39012717

[33] Ultragenyx Pharmaceutical/Mereo BioPharma. Phase 3 ORBIT and COSMIC results for setrusumab (UX143) in osteogenesis imperfecta. Press release. 2025. Available at: https://ir.ultragenyx.com/news-releases/news-release-details/ultragenyx-announces-phase-3-orbit-and-cosmic-results-setrusumab.

[34] Patseadou M, Haller DM. Vitamin D in Adolescents: A Systematic Review and Narrative Synthesis of Available Recommendations. J Adolesc Health. 2020;66(4):388–407. 10.1016/j.jadohealth.2019.08.025. 31685374

[35] Corsello A, Spolidoro GCI, Milani GP, Agostoni C. Vitamin D in pediatric age: Current evidence, recommendations, and misunderstandings. Front Med (Lausanne). 2023;10:1107855. 10.3389/fmed.2023.1107855. Published 2023 Mar 16. PMC10060648 37007781

[36] Yilmaz G, Hwang S, Oto M, et al. Surgical treatment of scoliosis in osteogenesis imperfecta with cement-augmented pedicle screw instrumentation. J Spinal Disord Tech. 2014;27(3):174–80. 10.1097/BSD.0b013e3182624b76 24945295

[37] Girdler SJ, Lindsey MH, Sebastian AS, Nassr A. Osteoporosis Evaluation and Management in Spine Surgery. J Am Acad Orthop Surg. 2024;32(18):e909–18. 10.5435/JAAOS-D-24-00311 39083525

[38] Chan E, DeVile C, Ratnamma VS. Osteogenesis imperfecta. BJA Educ. 2023;23(5):182–8. 10.1016/j.bjae.2023.01.005 PMC10140476 37124171

